# Role of Diet in Influencing Rheumatoid Arthritis Disease Activity

**DOI:** 10.2174/1874312901812010019

**Published:** 2018-02-08

**Authors:** Humeira Badsha

**Affiliations:** Dr. Humeira Badsha Medical Center, Beach Park Plaza, Jumeira Road, Dubai, UAE

**Keywords:** Diet, Rheumatoid arthritis, Microbiome, Inflammation, Dysbiosis, Disease

## Abstract

**Background:**

Patients with Rheumatoid Arthritis (RA) frequently ask their doctors about which diets to follow, and even in the absence of advice from their physicians, many patients are undertaking various dietary interventions.

**Discussion:**

However, the role of dietary modifications in RA is not well understood. Several studies have tried to address these gaps in our understanding. Intestinal microbial modifications are being studied for the prevention and management of RA. Some benefits of vegan diet may be explained by antioxidant constituents, lactobacilli and fibre, and by potential changes in intestinal flora. Similarly, Mediterranean diet shows anti-inflammatory effects due to protective properties of omega-3 polyunsaturated fatty acids and vitamins, but also by influencing the gut microbiome. Gluten-free and elemental diets have been associated with some benefits in RA though the existing evidence is limited. Long-term intake of fish and other sources of long-chain polyunsaturated fatty acids are protective for development of RA. The benefits of fasting, anti-oxidant supplementation, flavanoids, and probiotics in RA are not clear. Vitamin D has been shown to influence autoimmunity and specifically decrease RA disease activity. The role of supplements such as fish oils and vitamin D should be explored in future trials to gain new insights in disease pathogenesis and develop RA-specific dietary recommendations.

**Conclusion:**

Specifically more research is needed to explore the association of diet and the gut microbiome and how this can influence RA disease activity.

## 1 INTRODUCTION

Despite advances in the treatment of Rheumatoid Arthritis (RA), remission rates are still low [1]. Approximately 50% of patients were experimented with unorthodox treatments and diets in the recent 6 months probably in an attempt to gain better disease control [2]. There are clear links between diet and celiac disease but it is more difficult to prove these links in RA and osteoarthritis. Patients look to their rheumatologists for advice regarding dietary modifications and what they are told is that there is limited scientific proof regarding the effectiveness of a dietary approach aimed to help RA [3]. However, our recent understanding of the role of the gut microbiome in influencing RA disease activity has increased, leading to postulations that diet can influence the gut bacteria and hence RA disease activity.

The link between the gastrointestinal microbiome and immune system is well established and one can influence the activity of the other [4-6]. Gut microbiota plays an important part in the pathogenesis of systemic inflammatory diseases like RA. The influence is potentially mediated by altered epithelial and mucosal permeability and immune tolerance to the indigenous microbiota. Studies have shown that there is trafficking of activated immune cells to the

joints and altered expression of antigenic foci in the joints [7]. Filamentous bacteria in the gut have been reported to drive autoimmune arthritis through their influence on the T helper 17 cells [8]. Faecal microbiota has been characterized in (RA) and evidence supports the participation of intestinal microbes in the pathogenesis of RA [9, 10]. Changes in the microbiome due to changing lifestyle and dietary habits have led to immunological imbalances. This dysbiosis can influence the immunological damage in conditions like RA and inflammatory bowel disease [11].In patients with obesity, gut microbiota enhance autoimmune conditions by both direct and indirect mechanisms. The former are mediated by endotoxin-induced inflammation and the latter by regulatory B and T cells and T helper 17 cells [12]. The gut microbiota has been reported to influence immunological homeostasis due to its proximity to the host’s intestinal immunity and the ability to influence the immune responses. Dysbiosis impacts the innate immunological pathways which lead to the production of pro-inflammatory cytokines IL-1, IL-18, IL-17, IFNϒ, TNF, and others. The signalling pathways involve an array of molecules, *e.g.* serum amyloid A, CC-chemokine ligand 5, and others that are produced by autoreactive T cells. The autoreactive T cells also migrate to the peripheral immune compartments to activate the B cells to form antibody producing plasma cells. The antibodies and the plasma cells then migrate to the synovial tissues to initiate an inflammatory cascade. Macrophages, fibroblasts, osteoclasts, and proteinases are the key components of the synovial inflammatory processes [13].

Chronic inflammation promotes endothelial cell activation and vascular dysfunction that can induce atheroma formation in patients with RA. The putative mediators of atherogenic mechanisms include cytokines like TNF, IL-1, IL-17 and acute phase reactants like C reactive protein and serum amyloid P-component. These mechanisms potentially explain the higher risk of cardiovascular diseases in patients with RA [14]. Another shared inflammatory mechanism in vascular dysfunction and RA include the increased numbers of platelets and high levels of platelet microparticles (PMPs). The PMPs induce synovial fibroblast cytokine response to amplify the inflammation in RA [15].

With the increased understanding of microbiome in systemic inflammatory diseases like RA, dietary interventions and probiotics may find application in the management of such conditions. Dietary changes can impact the human intestinal microbiome leading to local inflammation and increased permeability. This can cause a systemic spread of pro-inflammatory lymphocytes and cytokines leading to inflammation in distant sites such as the joints [16]. Diet and weight loss are said to alleviate the disease burden in RA [17]. Microbial manipulation by dietary modifications, probiotics, antibiotics, and disease-modifying agents seems to modulate the disease process and progression in RA. In this review, the available evidence is evaluated for the potential role of diet in RA.

## 2 DIET IN RA

### 2.1 Mediterranean Diet and RA

Mediterranean diet is largely based on consumption of foods derived from plant sources with limited meat consumption. The main source of fats is small amounts of wine and olive oil. The role of Mediterranean diet in rheumatic diseases derives from anti-inflammatory and protective properties of omega-3 polyunsaturated fatty acids and vitamins. Constituents of olive oil such as oleic acid and olecanthal have natural anti-inflammatory properties [18].

There is conflicting evidence for the benefits of Mediterranean diet in RA. Some studies have reported a decrease in pain and disease activity with Mediterranean diet in RA [19]. In a 12 week randomised trial in 51 patients with RA, Mediterranean diet intervention demonstrated reduction in disease activity (measured by the 28 joint count Disease Activity Score (DAS28)), improvement in physical function (Health Assessment Questionnaire (HAQ)) and increased vitality [20]. These results can partly be attributed to antioxidant-rich foods during the Mediterranean diet [21].

During 3511050 person-years of follow-up in 83,245 and 91,393 women from the Nurses' Health Study (NHS; 1980-2008) and the NHS II (1991-2009), respectively, 913 participants developed RA. There was no significant association between the adherence to Mediterranean dietary pattern and the risk of RA [22]. In 208 patients from the TOMORROW study, high Monounsaturated Fatty Acids (MUFA) intake was an independent predictor of remission in the RA group with borderline boundary significance (odds ratio, 1.97; 95% CI, 0.98-3.98; P = 0.057) [23].

### 2.2 Flavanoids/Isoflavones and RA

Flavonoids are a group of phenolic compounds that are widely distributed in plants and fungus. The antioxidant, antimicrobial, and anti-inflammatory properties of flavanoids explain the role of these agents in atherosclerosis, rheumatoid arthritis, and other inflammatory conditions. The exact mechanisms for benefits of flavanoids in RA are unclear [24]. Genistein, the major active compound from soybean, has anti-inflammatory, anti-angiogenesis, anti-proliferative, antioxidant, immunomodulatory, pain relief, and joint protection properties. In vitro and in vivo studies suggest genistein to be a promising agent for the treatment of RA [25]. Genistein is shown to inhibit IL-1β, TNF-α or EGF-induced proliferation and MMP-9 expression in fibroblast-like synoviocytes of RA [26]. Additionally, genistein is shown to inhibit pro-inflammatory cytokines in human mast cell activation through the inhibition of the ERK pathway [27] and improve the serum paraoxonase activity and lipid profiles in rats [28].

### 2.3 Gluten Free Diets in RA

Gluten, a gut-derived antigen, is an immunological trigger in coeliac disease and RA. The systemic immune response in celiac disease may be directed towards sites other than the gut. Gut-derived antigens are the key initiators and drivers of dysimmunity in RA. These shared immunological mechanisms explain the concomitant occurrence of celiac disease and RA [29]. Gluten-free diet has been associated with benefits in patients with RA though the existing evidence is inconclusive. Gluten-free vegan diet for 1 year has been shown to significantly reduce levels of antibodies to β-lactoglobulin and gliadin and disease activity in patients with RA [30]. In a randomized study in 66 patients with RA, a gluten-free vegan diet demonstrated potentially atheroprotective and anti-inflammatory changes, including decreased LDL and oxLDL levels and raised natural atheroprotective antibodies against phosphorylcholine [31].

### 2.4 Elemental Diet and RA

An elemental diet contains amino acids (no whole proteins), mono/di-saccharides and medium/long-chain triglycerides. These are supplemented with vitamins and trace elements. In a pilot study, 30 patients with active rheumatoid arthritis were randomly allocated to 2 weeks of treatment with an elemental diet (n = 21) or oral prednisolone 15 mg/day (n = 9). In this study, elemental diet was as effective as a course of oral prednisolone 15 mg daily in improving subjective clinical parameters in RA except the swollen joint score. An improvement of greater than 20% in early morning stiffness, pain (VAS score) and the Ritchie articular index occurred in 72% of the elemental diet group and 78% of the prednisolone group [32]. However, not all patients benefit with elemental diet and this may be reserved for only select patients with RA [33].

### 2.5 Vegan and Elimination Diets and RA

Some studies have demonstrated benefits of vegan diet in RA [34] The benefits of vegan diet may be explained by antioxidant constituents, lactobacilli and fibre [35] and by potential changes in intestinal flora [36] In a single-blind dietary intervention study, 24 patients with moderate-to-severe RA reported significant reductions in symptoms with a 4-week, very low-fat (approximately 10%), vegan diet. At 4 weeks, weight (p < 0.001) and all measures of RA symptomatology decreased significantly (p < 0.05), except for duration of morning stiffness (p > 0.05). There were no significant reductions in C-reactive protein and RA factor while erythrocyte sedimentation rate remain unchanged (p > 0.05) [37].

In a study by Kjeldsen-Kragh (1999) [38], patients were assigned to fasting followed by a vegan diet; 12 of the 27 patients in the experimental diet group showed significant clinical improvement compared with only 2 of the 26 patients in the control group (P < 0.003, Fisher's exact test). After the study, patients were evaluated after 1 year. The diet responders continued to be statistically better than the non-responders in clinical variables.

There is insufficient evidence for absolute elimination of dairy products in the diet of patients with RA given the established benefits of dairy products for health [39]. Total elimination of dairy from diet is not recommended for control of symptoms in RA [40]. However, there is conflicting evidence for continued use of dairy products. Panush (1991) [41] demonstrated temporary improvement in the signs and symptoms of RA with diet elimination and modification in a controlled study where the symptoms associated with food sensitivities were studied. During this study, when the patients were fasting or on a severely restricted diet, the patients’ symptoms improved significantly. However, when the patients had milk reintroduced into the diet, episodes of pain, swollen and tender joints and stiffness were experienced.

### 2.6 Polyunsaturated Fatty Acids and RA

Long-term intake of fish and other sources of long-chain n-3 polyunsaturated fatty acids (n-3 PUFAs) have been reported to be protective for development of RA. In a prospective study in 205 women with RA, dietary intake of long-chain n-3 PUFAs (>0.21 g/day) was associated with a 35% decreased risk of developing RA (multivariable adjusted relative risk (RR) 0.65; 95% CI: 0.48 to 0.90) compared with a lower intake. Long-term intake of consistently >0.21 g/day was associated with a 52% (95% CI: 29% to 67%) decreased risk and consistent long-term consumption (fish ≥1 serving per week compared with<1) was associated with a 29% decrease in risk (RR 0.71; 95% CI: 0.48 to 1.04) [42].

Some studies support the role of ω-3 PUFAs supplementation as a valuable therapeutic option to improve symptoms, tender joint count, duration of morning stiffness, and the requirement of NSAIDs in RA [43]. In a recent meta-analysis of 20 randomized control trials (n=717), ω-3 polyunsaturated fatty acids reduced the level of leukotriene B4 (five trials, Standardized Mean Difference [SMD], -0.440; 95% confidence interval [CI], -0.676 to -0.205; I2=46.5%; P <0.001) and blood triacylglycerol levels (three trials, SMD, -0.316; 95% CI: -0.561 to -0.070; I2=0.0%; P=0.012) in RA [44].

### 2.7 Probiotics and RA

Current evidence supports the role of probiotics as adjunctive therapy in RA [45]. In a randomized double-blind clinical trial, 60 women with established RA received *Lactobacillus casei* 01 or placebo for 8 weeks. This study confirmed the role of *Lactobacillus casei* 01 as an adjunct therapy to help alleviate symptoms and improve inflammatory cytokines in RA. The probiotic supplementation decreased serum high-sensitivity C-reactive protein levels, tender and swollen joint counts, global health score, and DAS28 (P < 0.05). At the end of the study, a significant difference was observed between the intervention and placebo groups for IL-10, IL-12 and TNF-α changes (P <0.05) in favour of the probiotic group. Probiotic treatment was not associated with any adverse effects [46].

However, clinical trials have not demonstrated any consistent and appreciable impact on patient reported or laboratory outcome measures [47]. In a randomized double-blind placebo-controlled clinical trial in 60 patients with RA, daily supplementation with *Lactobacillus casei* 01 demonstrated no improvement in serum lipids [48]. In another study, *Lactobacillus casei* 01 demonstrated no significant impact on the oxidative status in patients with RA characterized by serum malondialdehyde, total antioxidant capacity, erythrocyte superoxide dismutase, and catalase [49].

A meta-analysis of 9 studies (n=361), established a reduction in levels of pro-inflammatory cytokines IL-6 with probiotics vs. placebo in RA (standardized mean difference: - 0.708; 95% CI: - 1.370 to 0.047, P=0.036). However, there was no difference between probiotics and placebo in disease activity score (mean difference 0.023; 95% CI - 0.584 to 0.631, P=0.940). The clinical effect of probiotics in RA remained unclear. The authors concluded that high-quality randomized controlled trials were needed to prove the effect [50]. Probiotics make an appealing therapeutic strategy in RA due to possible interactions with the microbiome but the effects need to be confirmed in interventional studies [51].

Recent studies show that specific bacteria may benefit RA patients. In a study in VDQ8 mice treatment with *P. histicola* in prophylactic or therapeutic protocols exhibited significantly decreased incidence and severity of arthritis compared to controls [52]. The microbial mucosal modulation of arthritis was dependent on regulation by CD103+ dendritic cells and myeloid suppressors (CD11b+Gr-1+ cells) and by generation of Treg cells (CD4+CD25+FoxP3+) in the gut, resulting in suppression of antigen-specific Th17 responses and increased transcription of interleukin-10. Treatment with *P. histicola* led to reduced intestinal permeability by increasing expression of enzymes that produce antimicrobial peptides as well as tight junction proteins (zonulaoccludens 1 and occludin).However, the innate immune response *via* Toll-like receptor 4 (TLR-4) and TLR-9 was not affected in treated mice.

There is evidence for alteration in gut microbiome with diets. In an analysis of stool samples from [53] RA patients, significant alteration in the intestinal flora was observed when the patients changed from omnivorous to vegan diet and for periods with vegan and lactovegetarian diets. The faecal flora from patients with high and low improvement indices differed significantly from each other at 1 and 13 months during the diet [53]. This finding of an association between intestinal flora and disease activity may have implications for our understanding of how diet can affect RA.

### 2.8 Alcohol and RA

Increased frequency of alcohol consumption is associated with severity of disease in RA. Alcohol has unfavourable effects on CRP, DAS28-CRP, pain VAS, modified HAQ, and radiographic damage (Larsen score) in RA [54]. Similar associations were reported in a prospective observational study in 615 patients of RA [55]. On the contrary, in 386 patients from the Västerbotten Intervention Program (VIP) cohort, alcohol was not associated with a risk of RA [56].

The risks for hepatotoxicity due to alcohol may be increased in patients with RA who are treated with methotrexate [57]. However, weekly alcohol consumption of <14 units does not appear to be associated with an increased risk of transaminitis in these patients [58]. The current evidence suggests that patients with RA should eliminate or limit alcohol consumption to very small quantities. The benefit and safety of moderate alcohol intake needs to be evaluated in clinical studies.

### 2.9 Vitamin D Supplements and RA

The relationship of vitamin D and RA is complex as the vitamin has a proven role in the immunological milieu in the pathophysiology of RA. Deficiency of vitamin D is common in patients with RA [59]. The COMORA Study confirmed the low levels of vitamin D in RA (n=1413, 15 countries) in different countries and in different latitudes. Low levels of vitamin D were associated with increased disease activity and corticosteroid dosage, and comorbidities in RA [60]. However, vitamin D supplementation does not have a disease modifying effect in RA. In a 12 week randomized, double-blind, placebo-controlled trial of 25-hydroxy vitamin D 50,000 IU weekly vs. placebo (n=117) in RA, supplementation with vitamin D did not affect the disease activity in RA [61].

Vitamin D has potent anti-inflammatory effects possibly due to suppression of IL-17 + and IFNγ+ T cells implicated in RA. However, the T cells in synovial fluid in RA have demonstrated a reduced sensitivity to vitamin D. Restoration of response to vitamin D in the memory T cells in synovial fluid represents a possible treatment strategy to be explored in RA [61]. Patients with active RA under steroid treatment may need supplementation with calcium and vitamin D to prevent osteoporosis [62]. and to alleviate the symptoms of anxiety and depression [63].

### 2.10 Other Supplements and RA

Iron has an important role in maintaining normal immune function and deficiency of iron and anaemia may hamper the immunological balance in RA [64]. Complex interactions of the pro-inflammatory cytokine, interleukin-6, the iron regulatory hormone, hepcidin, and the iron exporter, ferroportin underlie the impaired iron homeostasis in anaemia associated with inflammation [65]^.^ Correction of anaemia can help improve the physical activity and quality of life in patients with rheumatic diseases [66].

### 2.11 Anti Oxidants and RA

There is a marked increase in oxidative stress in RA. Patients with RA show marked increase in the formation of reactive oxygen species, lipid peroxidation, protein oxidation, DNA damage and decrease in the activity of antioxidant defence systems [67]. However, there is conflicting evidence in literature for the benefits of anti-oxidant supplementation in RA.

In a study in 40 women with RA, daily supplementation with 50 μg selenium, 8 mg zinc, 400 μg vitamin A, 125 mg vitamin C, and 40 mg vitamin E improved clinical outcomes and alleviated oxidative stress in RA. In this study, there was a significant improvement in disease activity but not in the number of painful and swollen joints. The antioxidant levels in erythrocytes were increased [68]. In another study, supplementation with the anti-oxidant vitamins such as vitamin A and C, or with trace elements like selenium and zinc had no proven influence on the disease activity in RA [62].

There is a significant association of low serum selenium levels and RA [24]. Serum copper levels are reported to be positively correlated with disease activity in RA. Low serum concentrations of albumin, zinc, and selenium were independently related to disease activity index in an evaluation of trace elements in 110 patients with RA [69].

### 2.12 Fasting in RA

A well balanced diet is important to meet the nutritional requirements in RA. Reduction of pain and inflammation in RA has been established with fasting [70] although reversal of remission is also known following resumption of regular dietary habits [71]. The benefits with fasting in RA, if any, are transient and may not have long-term implications in disease activity [72].

### 2.13 Obesity and RA

Obesity has been reported to decrease the odds of achieving and sustaining remission in RA. When compared to the non-obese, obese patients with RA have worse Disease Activity Scores or Disease Activity Scores in 28 joints, tender joint counts, inflammatory markers, patient global evaluation scores, pain scores, and physical function scores. Though obesity has not been associated with increased mortality in RA, interventions to prevent and reverse obesity can help to improve the outcomes and quality of life in RA [73]. Foods linked with obesity such as sweetened soda have been associated with an increased risk of RA. When compared to no consumption of sugar-sweetened soda or <1 soda per month, consumption of ≥1 sugar-sweetened soda per day has 63% increased risk of developing seropositive RA. [74]

## 3 CONCLUSION

Studies of dietary interventions for RA are limited and not of high quality. Most have heterogeneity in interventions and outcomes, baseline imbalance and inadequate data reporting [75].

Clinical trials of dietary treatments are difficult to design and implement due to problems associated with recruitment, compliance, and drop-outs. Recruitment in trials with dietary interventions is difficult, as it requires patients to change their lifestyles. Adherence to diet for a prolonged period may be difficult and trials with dietary interventions have a high dropout rate. Education about the diet and follow up in such trials requires dedicated efforts of a dietician. Challenges in design of dietary interventions include the process of blinding. Studies with long-term follow up (beyond 1 year) are difficult to implement and are lacking for diet in RA. [76] Studies should be planned and adequately powered to control for variables that can influence disease activity in RA including medications. Adjustments for confounding factors like smoking, physical activity, socioeconomic status, and obesity is important for validity of study results.

Additionally there is heterogeneity in tools used for assessments of dietary patterns in RA. Studies have reported the use of different questionnaires like the Brief Self-administered Diet History Questionnaire (BDHQ) [23] and the validated food frequency questionnaires [22]. Comparisons across studies may be difficult due to these trends. Dietary records for dietary intake are difficult to maintain and are subject to recall bias.

### 3.1 Gaps in Understanding and Directions for Future Research

There is currently a lack of understanding for nutritional requirements in RA. Specific dietary recommendations have not evolved due to variability in the clinical course of RA. In 386 patients from the Västerbotten Intervention Program (VIP) cohort, diet was not associated with a risk of RA. [55] In a recent feasibility trial, individualized counselling did not significantly improve the dietary habits in patients with inflammatory RA (n=31) of <1 year duration [77].

Future trials may evaluate specific dietary modifications, *e.g.* the association with moderate intake of alcohol and the role of probiotics and antibiotics in RA. There is a potential role of adipokines in mediating the association of RA and obesity. If studied in future trials, this can help to understand newer molecular targets for management of RA [12] Studies that link diet with RA and specific pathways of inflammation and immune regulation offer the possibility of identifying new therapeutic approaches in select patients. Better understanding of dietary factors in RA can contribute to developing new insights in disease pathogenesis and RA-specific recommendations.

The effects of ω-3 PUFAs supplementation on radiographic progression and synovial histopathology in RA remain to be evaluated in future studies. The role of polyunsaturated fatty acids in early arthritis and in combination with biologics should also be explored in well-designed trials in RA patients [43].

In summary, although we tell our patients there is a limited role for diet in RA, certain diets may help some groups of patients. However, the available evidence does not establish dietary interventions as a substitute for pharmacotherapy in RA. Limited or cyclical fasting, vegan, Mediterranean diets or elimination of dairy and gluten, all seem to play a role but it is hard to sustain these diets long term. Dysbiosis is a key factor in RA and diet can influence the gut microbiome and perhaps RA disease activity be manipulated through diet and alteration of gut bacteria. Probiotics could have a role although evidence for this is limited. Supplements with vitamin D, calcium, or fish oils could be useful where indicated.
